# Low back pain in junior Australian Rules football: a cross-sectional survey of elite juniors, non-elite juniors and non-football playing controls

**DOI:** 10.1186/1471-2474-12-159

**Published:** 2011-07-13

**Authors:** Wayne Hoskins, Henry Pollard, Chris Daff, Andrew Odell, Peter Garbutt, Andrew McHardy, Kate Hardy, George Dragasevic

**Affiliations:** 1 Department of Chiropractic, Macquarie University, Sydney, NSW 2109, Australia; 2 Harbord Village Chiropractic, Freshwater, NSW 2096, Australia; 3 Norwest Orthopaedic and Sports Physiotherapy, Norwest, NSW 2153, Australia; 4 Enhance Chiropractic and Massage Sports Injury Centre, Ngunnawal, ACT 2913, Australia

## 

The journal has been informed by the authors' institution that, contrary to the statement in this article [1], the Macquarie University Human Ethics Committee did not receive an application for ethics approval for this study. As the study was conducted without institutional ethics committee oversight, this article has been retracted.

## Pre-publication history

The pre-publication history for this paper can be accessed here:

http://www.biomedcentral.com/1471-2474/12/159/prepub
